# The influence of bitter-taste receptor (TAS2R) expression in pharmacological response to Chloroquine in obese patients with COVID-19

**DOI:** 10.6061/clinics/2020/e2181

**Published:** 2020-08-21

**Authors:** Lígia Moriguchi Watanabe, Izabella Ferreira Pires, Natália Yumi Noronha, Marcela Augusta de Souza Pinhel, Carla Barbosa Nonino

**Affiliations:** I Departamento de Clinica Medica, Faculdade Medicina de Ribeirao Preto (FMRP), Universidade de Sao Paulo, Sao Paulo, SP, BR.; II Departamento de Biologia Molecular, Faculdade de Medicina de Sao Jose do Rio Preto (FAMERP), Sao Jose do Rio Preto, SP, BR.

The coronavirus disease 2019, referred to as COVID-19 by the World Health Organization (WHO), is an ongoing viral pandemic caused by the influenza-like virus strain SARS-CoV-2 (1). According to WHO (2020), SARS-CoV-2 infection has been responsible for approximately 10,185,374 confirmed cases of COVID-19 and more than 500,000 deaths worldwide (2). In Brazil, 1,368,195 cases have been confirmed, and 58,314 deaths have occurred as of June 30, 2020 (3).

The coronaviruses are one of the primary pathogens that target the human respiratory system. These viruses often cause pneumonia-like symptoms that can develop into Severe Acute Respiratory Syndrome (SARS), causing higher leukocyte production, abnormal respiratory findings, and increased levels of plasma pro-inflammatory cytokines (4). COVID-19 may manifest either as an asymptomatic infection or as a mild to severe pneumonia (5). The most common initial symptoms at the onset of COVID-19 are fever, cough, and fatigue, while sputum production, headache, hemoptysis, diarrhea, dyspnea, and lymphopenia are less frequent (6).

The severity of the COVID-19 infection varies among individuals and may relate to several reported risk factors, such as aging, diabetes mellitus, cardiovascular risk (including hypertension), and respiratory or kidney disease. Moreover, an increasing number of studies have linked obesity to more severe COVID-19 symptoms and, ultimately, increased mortality (7-9).

Recently, a retrospective analysis of 3,615 patients with COVID-19 at the New York academic hospital system reported that approximately 38% of these patients were obese (Body Mass Index, BMI >30 kg/m^2^). Moreover, obese patients under 60 years of age had twofold susceptibility to being admitted in critical care compared to non-obese individuals (8).

Obesity-related conditions worsen the impact of COVID-19 symptoms due to complications associated with excessive body weight, metabolic dysfunction, cardiovascular risk, sleep apnea, vitamin D deficiency, dysregulation of the renin-angiotensin-aldosterone system, and sarcopenia (10). Furthermore, obese patients have altered levels of circulating cytokines. Notably, these individuals exhibit higher concentrations of TNF-alpha, MCP-1, and IL-6, which are generated by visceral and subcutaneous adipose tissue (11). This alteration in inflammatory profile appears to predict the severity and prognosis of obese patients and COVID-19 (11,12). Thus, special attention should be considered for this population.

In the absence of efficient pharmacotherapy, and due to the public health emergency, a significant number of potential drugs was proposed, such as antiviral agents, chloroquine and hydroxychloroquine, and corticosteroids (13,14).

Chloroquine (CQ) is an amine acidotropic form of quinine. In the past, this compound was the drug of choice against malaria and has been reported to be a potential broad-spectrum antiviral drug (13). The mechanism of action of CQ and its analog hydroxychloroquine (HCQ) consists in blocking viral entry into cells by interacting with viral particles that bind to human cell surface receptors (15). Both CQ and HCQ can have immunomodulatory effects through the modulation of pro-inflammatory cytokines (13).

CQ is also an active agonist of bitter-taste receptors (TAS2Rs), first identified in the bud cells of the tongue. These receptors signal information to the brain about the nutritive value or toxicity of ingested foods and beverages (16,17). Aside from their role in chemosensory cells, these receptors are expressed in an extensive range of tissues and perform varied functions (18). A recent study conducted by Grassin-Delyle et al. (19) suggested that TAS2Rs 3, 4, 5, 9, 10, 14, 30, 39, and 40 were involved in the inhibition of cytokine production. Moreover, Li et al. (12) hypothesized that TAS2R10 might help to prevent the cytokine storm - a key event for patients with more aggressive COVID-19 symptoms - because its receptor regulates natural killer cell-mediated cytotoxicity, and chemokine, T-cell receptor, and TNF signaling pathways (12). CQ binding to TAS2Rs may also result in functional changes in respiratory tract cells, such as smooth muscle, ciliated cells, and blood cell markers. This suggests that the extra-oral effects of CQ could be beneficial against pulmonary diseases (20).

Interestingly, extra-oral TAS2R expression could be reduced in obese subjects compared to non-obese individuals (18). This may result in a decreased sensitivity to bitter taste, which is often observed in obese individuals, and may affect food choices (18). Considering the role of TAS2R and its agonists on immune responses, and the fact that CQ is a TAS2R agonist, we hypothesized that COVID-19-infected obese patients could respond differently to pharmacological treatment with CQ and side effects could occur more frequently due to overdosage in this population. This hypothesis is supported by a recent study that evaluated the risk of retinopathy - one of the most important long-term side effects of HCQ treatment - related to clinical characteristics and HCQ blood levels of 537 patients (21). The authors found a significant association between higher BMI and a higher risk of HCQ toxicity, suggesting a limited HCQ dosage at 400 mg daily, irrespective of how high is the patient’s BMI (21).

Moreover, according to Karalis et al. (22), dosage regimens of CQ/HCQ used for treating COVID-19 symtoms could be linked to high toxicity risk. Thus, to avoid adverse effects due to CQ/HCQ overdosage, the authors proposed dosage regimens tailored to the patient’s characteristics, such as body weight, age, and severity of COVID-19 symptoms (22). However, more studies are required to further address the influence of individual factors in the CQ/HCQ pharmacodynamics and the optimal dosage of CQ/HCQ for treating COVID-19 symptoms (15,23). Figure 1 summarizes the association of TAS2R expression and the response to CQ and HCQ of obese patients in the context of COVID-19 infection.

## CONCLUSION

The COVID-19 pandemic is a significant global public health challenge, especially concerning the pursuit of effective drugs for its prevention and treatment. Considering the prevalence of obesity worldwide and the complications associated with excessive body mass ratio, special attention should be given to patients with obesity. In a condition of obesity, reduced TAS2R expression could affect the response to medications such as CQ. Altogether, we highlighted the importance of obese patient care, particularly during the current COVID-19 pandemic.

## AUTHOR CONTRIBUTIONS

All of the authors participated in the discussion, and manuscript writing, review and approval of the final version.

## Figures and Tables

**Figure 1:**
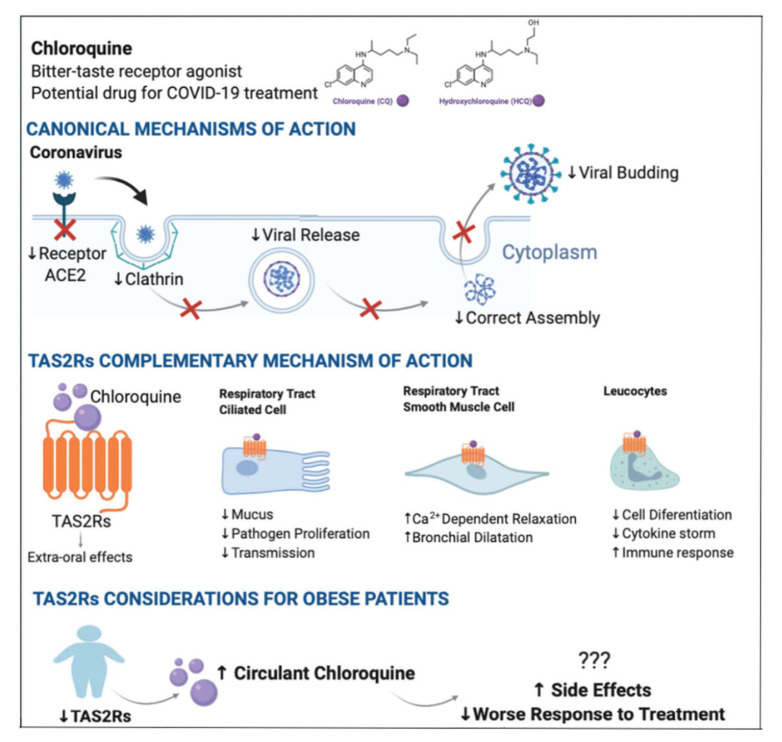
TAS2R molecular mechanisms in response to CQ and HCQ in obese patients on COVID-19 infection context.
